# Wellbeing After Finalization of a Workers’ Compensation Claim: A Systematic Scoping Review

**DOI:** 10.1007/s10926-023-10168-6

**Published:** 2024-01-30

**Authors:** James Weir, Robyn Fary, Mark Gibson, Tim Mitchell, Venerina Johnston, Mary Wyatt, Robert Guthrie, Bronwyn Myers, Darren Beales

**Affiliations:** 1 Faculty of Health Sciences, Curtin School of Allied Health, Curtin University, Perth, WA Australia; 2 Faculty of Health Sciences, Curtin enAble Institute and Curtin School of Allied Health, Curtin University, Perth, WA Australia; 3 Pain Options, 7 Hardy Street, South Perth, WA Australia; 4 Centre for Health Research, University of Southern Queensland, Darling Heights, Australia; 5 School of Health and Medical Sciences, University of Southern Queensland, Ipswich, QLD Australia; 6 Monash Centre for Occupational and Environmental Health (MonCOEH), Monash University, Melbourne, VIC Australia; 7 Faculty of Business and Law, School of Management and Marketing, Curtin University, Perth, WA Australia

**Keywords:** Wellbeing, Workers’ compensation, Workplace injury, Occupational health

## Abstract

**Objective:**

A workers’ compensation claim may have significant negative impacts on an injured worker’s wellbeing. Wellbeing provides a good global measure of potential effects of a claim on an individual, and is important for contemporary economic modelling. The purpose of this study was to synthesize knowledge about the wellbeing of injured workers after the finalization of a workers’ compensation claim and identify gaps in the current literature.

**Methods:**

A systematic scoping review was conducted.

**Results:**

71 full-text articles were screened for inclusion, with 32 articles eligible for this review. None of the included articles evaluated overall wellbeing. Included articles did evaluate a variety of constructs inherent in wellbeing. Injured workers were generally disadvantaged in some manner following claim finalization. The literature recommends a focus on reducing negative impacts on injured workers after finalization of a compensation claim, with a need for regulatory bodies to review policy in this area.

**Conclusion:**

There appears to be potential for ongoing burden for individuals, employers, and society after finalization of a workers’ compensation claim. A gap in knowledge exists regarding the specific evaluation of wellbeing of injured workers following finalization of a workers’ compensation claim.

**Supplementary Information:**

The online version contains supplementary material available at 10.1007/s10926-023-10168-6.

## Introduction

### Workers’ Compensation Systems Assist Most Injured Workers Return to Work

Good work is beneficial for health and wellbeing [1, 2]. The primary purpose of most modern workers’ compensation schemes is to support injured workers financially and/or with medical care following a work-related injury or disease. Workers’ compensation systems may also assist injured workers to return to their pre-injury level of work, before considering alternative pathways such as suitably modified or alternative employment. In the 2019–2020 reporting period in Australia, 115,707 serious injury claims involving at least one working week-off work were accepted [3]. The burden of these injuries is evident in the duration of a workers’ compensation claim, which was 537 days on average, between 2019 and 2021 in Australia [4]. Despite this, most injured workers in Australia (81.3%) returned to some degree of work following their injury and continued to be in paid employment of some description [5], which is consistent with other similar jurisdictions internationally [6, 7]. This also is indicative of approximately 20% of injured workers, who do not return to paid employment following their injury.

### Workers’ Compensation Claims May Negatively Influence Wellbeing

**Fig. 1:**
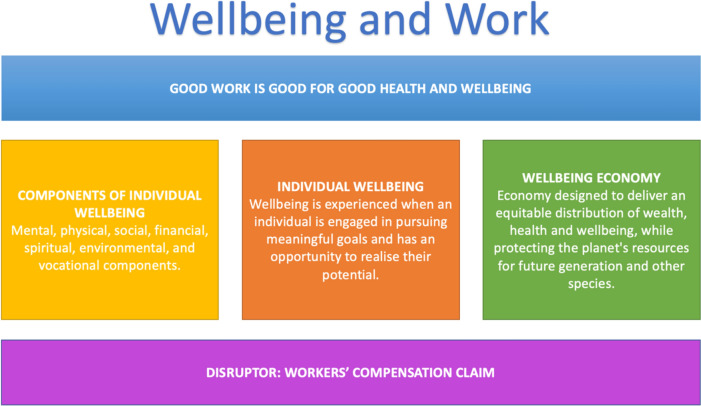
Constructs related to wellbeing and work

Wellbeing is experienced when an individual is engaged in pursuing meaningful goals and has an opportunity to realize their potential [8]. The World Health Organization states that “Well-being encompasses quality of life and the ability of people and societies to contribute to the world with a sense of meaning and purpose” [9]. Importantly, it acknowledges not only the negative aspects of ill health, but also the positive aspects of living. In this way, wellbeing at the individual level can be broadly considered as encompassing our physical and mental health, relationships, the quality of our work and job satisfaction, political and spiritual freedom, and our physical environment [10] (Fig. 1). The concept of wellbeing is also becoming more important for contemporary economic modelling (the wellbeing economy) [11] (Fig. 1).

Participation in a workers’ compensation claim can negatively affect wellbeing in multiple ways [12, 13]. Aurbach [14] posits that exposure to a compensation claim can result in altered, unhelpful behaviours and “needless disability”. Caruso [15] describes that “worklessness” is associated with increased morbidity and mortality, as well as negative psychological, social, and economic effects on an individual, their family, and their community [15]. Other systemic impacts during a claim can include medicalization (the process through which some aspects of human life, which were not previously pathologized, come to be considered as medical problems) [16], and iatrogenesis (the intentional or unintentional harm that comes to a person through the action or inaction of a health care provider or health delivery system). This may be immediate or delayed, yet preventable or even avoidable [17]. Complex administrative systems, independent examinations, legal representation, and adversarial environments are elements of a workers’ compensation claim associated with loss of control, which can have a negative impact on wellbeing [14, 18, 19]. Ultimately these issues might result in sub-optimal functional and psychological outcomes [20]***.*** A systematic review of 29 studies from a range of international compensation jurisdictions supports this view, describing an association between having a claim and poorer physical and psychological functioning [21]. An evaluation on the effects of financial compensation for workplace injuries in Australia reported that injured workers receiving workers’ compensation were vulnerable to financial stress [22]. Kilgour et al. [23] also identified in a systematic review of qualitative studies, a “growing consensus that involvement in compensation systems contributes to poorer outcomes for claimants” (p. 160). Workplace culture and satisfaction, as perceived by the injured worker, can also negatively influence return to work outcomes following workplace injury [24]. Management of musculoskeletal conditions is associated with poorer outcomes in workers’ compensation settings [14, 15, 25]. For injured workers who do experience negative influences during their compensation claim, it is plausible that their wellbeing might continue to be affected beyond the finalization of the claim [7].

### It is Not Clear What Happens to Individuals Following Claim Finalization

There are a range of different modes of claim finalization which vary with the jurisdictions within and between countries. Typically, claim finalization may be implemented for the following reasons: (1) an individual is deemed to have met the criteria for a successful medical recovery and returned to pre-injury employment or alternative employment; (2) the designated time limit for a compensable period is reached; (3) an agreement is reached between an individual and an insurer to exit the claim environment, and an appropriate negotiation for ongoing support (if applicable) outside the claim is undertaken; (4) an approved medical specialist assesses and deems that an individual has reached Maximum Medical Improvement which may be at a level less than full medical recovery, resulting in determination of a permanent impairment which may attract a prescribed or statutory amount of financial compensation; (5) a claim is finalized via legal avenues; or (6) compensation entitlements are exhausted. Different jurisdictions will utilize differing terminology to indicate the end-point of a compensation claim period. In some jurisdictions, a claim may be considered inactive, rather than settled or finalized. The needs and wellbeing outcomes of individuals returning to work and life once they leave the system are not well understood. Poorer post-claim outcomes may lead to further health and social burden on injured workers, and increased demands on health and social service/welfare systems [26–28].

The end of a workers’ compensation claim is not always associated with full recovery or return to work. Negative outcomes for employment and function have been noted even up to 5 years following claim settlement [29]. In addition, there is some indication that post-claim outcomes might be worse for those who are already socially disadvantaged [30, 31]. In general, however, literature investigating the health, financial, and employment status of individuals after finalization of a claim appears limited, and there is certainly no consolidated body of knowledge on this topic. Wellbeing is an important measure for community-based social support systems such as workers’ compensation [32, 33], but post-claim wellbeing outcomes are unclear. Therefore, the purpose of this study was to review the literature in relation to wellbeing and other associated outcomes of individuals following finalization of a workers’ compensation claim and identify gaps in the literature related to this.

## Methods

### Design

A systematic scoping review was deemed a suitable methodology for this study [34]. The review aimed to identify the types of available evidence related to the purpose of the study, to examine how research is conducted in this field, to describe key characteristics of wellbeing and other outcomes following finalization of a workers’ compensation claim and to identify gaps in our understanding of life following a workers’ compensation claim. Established frameworks for scoping reviews were used to inform the processes taken in completing the review [35, 36]. The review has been reported in accordance with the Preferred Reporting Items for Systematic Reviews and Meta‐Analyses Extension for Scoping Reviews (PRISMA‐ScR) [37]. Prior to undertaking the database search, the Scoping Review protocol was submitted for registration on the Joanna Briggs Institute Review Register (https://jbi.global/systematic-review-register, registration date 17th March 2022).

### Types of Participants

The participants of interest were adults aged over 18 years, who had engaged in, and subsequently finalized a workers’ compensation claim or ceased engagement with the insurer. As different jurisdictions utilize varied terminology or system-based definitions to delineate the end-point of a workers’ compensation claim process, a range of terminologies were considered in the search strategy (Appendix 1).

### Concept

The concept of the scoping review was to understand the experience of individuals following finalization of a workers’ compensation claim. Wellbeing was the primary focus of interest because of the potential effect of a workers’ compensation claim extending beyond impacts on physical capability, mental health status, and health-related quality of life. Also, there is increased recognition of the importance of wellbeing as a multi-dimensional adjunction to traditional economic measures of social programs [38]. However, the research team anticipated that the literature on the specific concept of wellbeing was likely to be limited in the literature. Hence, individual constructs of wellbeing such as financial status, employment status, health status, quality-of-life status, and relationship status were incorporated in the search strategy (Appendix 1).

### Context

The context was settings where individuals had engaged in, and subsequently finalized or ceased engagement with, a workers’ compensation insurance claim in a jurisdiction with a legal and regulated workers’ compensation scheme.

### Sources

The review considered English language quantitative studies, qualitative studies, mixed-method studies, and online resources.

### Inclusion Criteria

Research that considered wellbeing, or constructs of wellbeing, in injured workers aged 18 years or older, following the finalization of a workers’ compensation claim in a regulated workers’ compensation scheme was included. All study designs and papers written in English were included for consideration.

### Exclusion Criteria

The literature was excluded if published in languages other than English, or if there was no clear indication of a workers’ compensation claim having ended. Conference papers, editorials, and review articles were excluded.

### Search Strategy

The initial step utilized the assistance of the Curtin University Librarian to develop a limited search of broad multidisciplinary databases including Scopus and Proquest as well as a medical database Medline (Pubmed). The aim was to discover literature where specific measures of wellbeing had been included, as well as literature that included individuals’ experiences and health or social outcomes following claim finalization. The abstracts were screened to evaluate relevant search terms or keywords that should be included in the final search. Once the final search terms were confirmed, a search was carried out in all included databases; Scopus, Proquest, Business Source Ultimate, Medline (Pubmed) and Web of Science. These databases were selected due to their broad, multidisciplinary natures, to allow for medical, social, and legal forums to be explored. The date range utilized was from 1884 (considered the inception of modern workers’ compensation insurance under Prussian leader Otto von Bismarck [39]) to November 2022.

Further hand searching of the reference lists of the 32 included articles was carried out to identify additional relevant published literature for inclusion. The scoping review also included internet searching, to identify online information sources, reports, media releases, or websites that considered wellbeing, or constructs of wellbeing in injured workers, following finalization of a workers’ compensation claim. The search terms were inputted verbatim into web-based searching using Google and Google Scholar. Further to internet-based searching, specific exploration of jurisdictional regulatory websites was also undertaken. This included the states of New South Wales and Victoria in Australia, Ontario in Canada, and Missouri, California, and Washington State in the United States of America, as well as Sweden and Taiwan, as these were the jurisdictions represented in the included articles. In addition to this, further internet searching of jurisdictional regulatory websites was undertaken in the United States of America, Canada and Australia.

### Study Selection Process

Suitable literature was exported to EndNote® before being assessed for duplicates and exported to Covidence® (http://www.covidence.org) for title and abstract screening. Abstract screening was completed independently by two researchers (JW and MG), to select appropriate literature, with any disagreements being resolved by a third reviewer (DB).

This was then followed by full-text review for suitability using Covidence® independently by two researchers (JW and MG) to select the final literature for inclusion, with any disagreements being resolved by a third reviewer (DB).

### Extraction of Results

A data extraction template was developed within the Covidence® software. Initial extraction on the first 20% of the articles was performed independently by two researchers (JW, MG) to check for consistency and suitability of the extraction process. JW, MG and DB reviewed the quality of the data being extracted, modified the extraction template to include participant characteristics and the time since claim finalization, and refined the labelling of the extracted data. The final data extraction framework included the study characteristics (design, geographical location, the injury type being evaluated), characteristics of the participants (number, time since claim finalization, age, sex), measures of overall wellbeing or other specific wellbeing constructs, key findings, and any recommendations made by the authors. JW completed extraction of all the data, with assistance and review from DB.

### Critical Appraisal

Critical appraisal is not an essential component of the scoping review process and in accordance with guidelines can be included as an optional step in the process [37]. In order to provide the reader with an understanding of the characteristics of the literature in this area, critical appraisal of the final 32 included articles was completed utilizing the Joanna Briggs Institute Critical Appraisal Tools, relevant to each study design. Despite being designed for use in systematic reviews, these tools are also endorsed as an educational tool [37]. These tools do not provide a grade of quality, rather serve to facilitate an understanding of methodology of the studies included in the review and are used at the discretion of the research team [40]. Critical appraisal was completed on 20% of the articles by two independent researchers (JW, MG). The level of agreement was validated by a third researcher (DB). JW then completed critical appraisal of the remaining articles.

## Results

### Summary of Articles

**Fig. 2:**
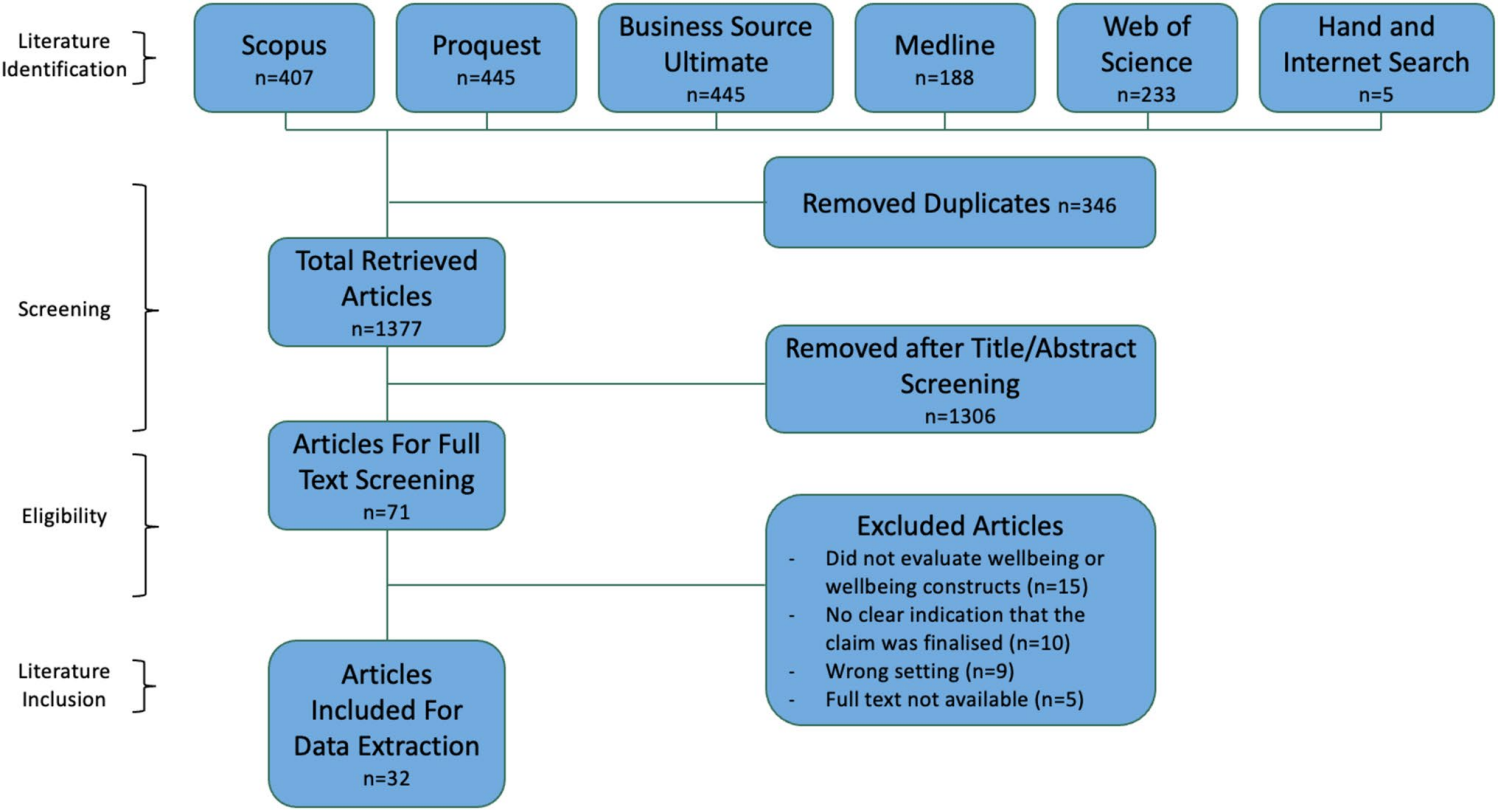
PRISMA flow chart

The results of the article inclusion process are depicted in Fig. 2. The data search retrieved 1723 articles, 5 of these from additional hand searching of the included articles and internet-based searching. Following removal of duplicates and title and abstract screening, 71 articles were included in full-text screening. Following full-text screening, a total of 32 articles were included for extraction [29–31, 41–69]. The articles ranged in time of publication from 1970 to 2022. A large proportion of the articles were completed by a small range of research teams and authors. Two people were authors on 8/32 articles (Chibnall and Tait). Another person (Sears) was an author on 7/32 articles, all 7 of which were published in 2021 and 2022. Terms used to denote claim finalization were permanent impairment/disability (26/32), settlement (14/32), claim/case closure (13/32), and lump sum (4/32). Data extracted from each study are provided in Table 1.

**Table 1:** General results table

Study	Study type, geographical location, and injury type	Participant demographics	Post-claim finalization outcome constructs	Main findings related to post-finalization outcomes	Recommendations related to post-finalization outcomes
Balla and Moraitis [37]	Clinical cohort study Melbourne, Australia Back or neck injuries	*n* = 82 Industrial accidents * n* = 59 Traffic accidents *n* = 23 (all participants of Greek origin) Time since finalization 1 to 3 months (*n* = 5) 4 to 6 months (*n* = 5) 7 to 12 months (*n* = 9) 13 to 24 months (*n* = 19) 24 months and over (*n* = 44) No delineation between work and traffic claims is made Age 21 and over Sex 31% Female, 69% Male	Symptoms Return to Work/Employment Status Satisfaction	32% of injured workers reported that their settlement was satisfactory 49% of injured workers had returned to work prior to settlement, 13% returned following settlement and 38% did not return to work at all	1. Further work is needed to determine exact mechanisms of pain and immobility 2. Early mobilization and specific measures aimed at the cause of pain may prevent problems that develop later 3. Measures to help people understand their new environment (after injury). should be identified 4. Further study is required to determine significant factors that influence the outcomes for each individual
Ballantyne et al. [38]	Work injury database cohort study Ontario, Canada Any work injury	*n* = 495 Time since finalization 6–52 months Age Mean = 45.52 Sex Female 61%, Male 39%	Wages and Financial	Being assessed with a permanent impairment increases the likelihood of future poverty for those injured workers who have substantially lower incomes and higher levels of personal poverty at the time of injury	1. Improve the identification of claimants who are in poverty 2. Improve the rights of injured workers who are permanently impaired
Casey and Ballantyne [39]	Work injury database cohort with comparison to a general population Ontario, Canada Any work injury	*n* = 494 *n* = 4486 for the comparative 2003 population sub sample *n* = 4495 for the comparative 2009/10 population sub sample Time since claim finalization 6–52 months Age Mean = 41 Sex Female 51%, Male 49%	Symptoms Physical Health/Physical Disability Mental Health	Injured workers with certified permanent impairment reported significantly more chronic health conditions than the general population. The most noted conditions with higher odds ratios in the work disability group included arthritis, intestinal or stomach ulcers, back problems and depression	1. An increased awareness of the negative health impacts of permanent impairment is recommended. This should include responses from health workers and the Workers’ Compensation Board
Chibnall et al. [27]	Workers’ compensation database cohort who underwent post-settlement interviews Missouri, USA Low-Back Injuries	*n* = 1475 African-American participants *n* = 580 Caucasian participants *n* = 892 Time since claim finalization Mean 21 months Age 18 and above Sex Female 38%, Male 62%	Symptoms Mental Health Wages and Financial Physical Health/Physical Disability	African-American and lower socioeconomic status injured workers had increased risk for poor post-settlement outcomes (higher levels of pain, greater psychological distress, more disability, and greater financial struggle) in comparison to Caucasian and injured workers with a higher socioeconomic status	1. Further research required on the issue of social equity in Workers’ Compensation systems 2. Mandates at federal and state levels required to assist social equity
Chibnall and Tait [40]	Workers’ compensation database cohort who underwent post-settlement interviews Missouri, USA Low-Back Injuries	*n* = 1475 African-American participants *n* = 580 Caucasian participants = 892 Time since claim finalization Mean 21 months Age 18 and over Sex Female 38%, Male 62%	Physical Health/Physical Disability Wages and Financial Satisfaction	For African-American and lower socioeconomic status injured workers in the Workers’ Compensation system, less treatment/compensation was associated with lower satisfaction with the process, which in turn predicted higher levels of post-settlement disability	1. Prospective, longitudinal studies are recommended related to the impact of satisfaction on post-settlement outcomes
Chibnall et al. [41]	Workers’ compensation database cohort who underwent post-settlement interviews Missouri, USA Low-Back Injuries	*n* = 475 African-American participants *n* = 580 Caucasian participants *n* = 892 Time since claim finalization Mean 21 months Age 18 and over Sex Female 38%, Male 62%	Return to Work/Employment Status	Injured workers who underwent disc-related surgery did not have any greater return to work disability after settlement than those who did not undergo surgery	1. Future research is recommended to facilitate equity in the management of occupational low-back injuries
Chibnall and Tait [28]	Workers’ compensation database cohort Missouri, USA Low-Back Injuries	*n* = 374 Time since claim finalization Mean 72 months Age Mean = 44 Sex Female 40%, Male 60%	Symptoms Mental Health Disability	Poorer long-term adjustment (defined as higher levels of pain, catastrophising, and pain-related disability) was predicted by lower socioeconomic status, race and poorer adjustment in the earlier stages after claim-finalization Overall outcomes were stable six years after claim closure when compared to more than 4 years earlier	1. Further understanding of the individual factors that influence long-term disability post-finalization is warranted 2. Recommends fundamental change to workers’ compensation system to promote equity in post-finalization outcomes, without providing further specific detail
Chibnall and Tait [42]	Workers’ compensation database cohort Missouri, USA Low-Back Injuries	*n* = 1464 No attorney group *n* = 220 Attorney group *n* = 454 Dissatisfied/attorney group *n* = 790 Time since claim finalization 21 months post-settlement n = 1464 72 months post-settlement *n* = 371 Age 18 and above Sex Female 38%, Male 62%	HRQol* Symptoms Mental Health Legal Proceedings Satisfaction	Post-settlement, the dissatisfied/attorney group reported socioeconomic stress and catastrophising The dissatisfied/attorney group reported higher levels of disability and catastrophising and lower levels of mental health status at longer-term follow-up	1. Further research is recommended to understand the reasons for attorney retention 2. Dissatisfied injured workers may require more specific attention during the course of the claim, to improve outcomes
Edmonds et al. [43]	Survey and administrative data of people from a workers’ compensation database Washington State, USA Any work injury	*n* = 442 Precarious employment *n* = 72 Less-precarious employment *n* = 370 Time since claim finalization Approximately 1 year Age 18 and above Sex Female 68%, Male 32%	Return to Work/Employment Status Physical Health/Physical Disability Reinjury Wages and Financial	After finalization of a claim with a permanent impairment, injured workers who were employed in non-standard or precarious jobs were more likely to have unsustained or different work return to work compared to those in full time employment Non-standard and precarious injured workers were also more likely to report poor health and wellbeing related measures after claim closure	1. Further research is recommended to facilitate understanding of the long-term health and employment impacts of non-standard and precarious jobs during workforce re-integration after injury 2. As non-standard and precarious jobs become increasingly common, these findings could inform federal and state vocational rehabilitation and transitional return to work efforts to help disabled injured workers with transitions into safe and secure employment
Evans et al. [44]	Clinical cohort undergoing a rehabilitation program Dallas, Texas, USA Low-back pain	*n* = 395 Recurrent injury group *n* = 172 Nonrecurrent injury group *n* = 223 Time since claim finalization Not defined, however the recurrent injury group had one prior claim Age Mean = 42.6 years Sex Female 36%, Male 64%	Mental Health Return to Work Health Care Utilization Return to Work/Employment Status	More injured workers with repeat low-back injuries reported pre-present injury psychopathology and substance abuse/dependency, more comorbidities and more job stability than the nonrecurrent group There was no difference for those with recurrent versus nonrecurrent injuries for response to rehabilitation and multiple socioeconomic outcomes including RTW, work retention, further treatment and surgery	1. Identifying and managing psychopathology in this population may help prevent future recurrence of injury
Foley and Silverstein [45]	Survey of workers from a workers’ compensation database Washington State, USA Carpal tunnel syndrome the primary injury of interest (with comparators of dermatitis and upper extremity fractures)	*n* = 1255 Carpal tunnel syndrome group *n* = 821 Upper extremity fractures *n* = 349 Dermatitis *n* = 85 Time since claim finalization Between 6 and 7 years Age Mean = 39.53 Sex Female 51%, Male 49%	Physical Health/Physical Disability Mental Health Personal Relationships Wages and Financial Return to Work/Employment Status	The burden of carpal tunnel syndrome includes continuing pain, loss of function, adverse financial impacts and household disruption which extend long after claim closure Health Domain Reduced function and increased medication use was more likely in the carpal tunnel syndrome group Social Domain Injured workers with carpal tunnel syndrome were less likely to be able to engage in normal household duties and their partners were more likely to need to increase their work hours. Carpal tunnel syndrome injured workers were also more likely to separate from their partners after the injury Economic Domain Injured workers with carpal tunnel syndrome were more likely to access social security supports or have reduced earnings following their claim Work Domain Injured workers with carpal tunnel syndrome were more likely to have reduced or unstable employment after their claim	1. The burden of carpal tunnel syndrome exists across a wide range of domains, not only wage replacement. This warrants further understanding and policy change to address these ongoing and multi factorial burdens
Greenough and Fraser [26]	Clinical cohort Adelaide, Australia Low-Back Injuries	*n* = 274 (67 with settled claims) Time since claim finalization Median = 22 months Age Range = 18 to 65 Sex Female 45%, Male 55%	Symptoms Physical Health/Physical Disability Mental Health Return to Work/Employment Status	Settlement of the claim did not influence employment status or psychological disturbance at follow-up compared to those who had not settled their claim at the time of follow-up, and these groups did not vary even up to 5 years post-settlement Following settlement, pain, disability and physical impairment were unchanged for men compared to those who had not settled their claim Following settlement, disability levels improved for women compared to those who had not settled their claim Reported reasons for lump sum claimants to not undergo claim procedure again: Too stressful It had caused family trauma Appeared to reduce treatment they were given They had become depressed Unable to subsequently find a job	1. The lump sum system of compensation for low-back injury should be abolished 2. Legal involvement should not occur 3. A continuous payment system should be considered for these cases
Harris et al. [46]	Clinical cohort presenting to a major metropolitan trauma centre Metropolitan New South Wales, Australia Major physical trauma with an injury severity score of more than 15	*n* = 355 (59 with settled claims, 48/59 with workers’ compensation claims) Time since claim finalization Mean = 19.2 months (however this does not discriminate between types of claims) Age Mean = 47.8 Sex Female 28%, Male 72%	HRQoL* Physical Health/Physical Disability Mental Health	Settling a claim had a positive effect on the SF-36 Mental Component Summary compared to having an unsettled claim	1. Recommend comparison of systems that differ in case-settlement times
Ho et al. [47]	Work injury database cohort with comparison to a general population Taiwan Any work injury	*n* = 81,249 insurance cases awarded permanent disability Time since claim finalization Up to 15 years following permanent disability Age Mean = 37.5 at time of injury Sex Not defined	Mortality	Permanent disability resulted in higher levels of mortality Mortality for those surviving the acute injury with resultant permanent disability was higher compared to the general population Mortality was relatively higher in young workers and female workers with a permanent disability	1. Call for a focus on prevention of work injuries to reduce the problem of increased mortality at the source 2. Recommend review of compensation payments for mortality and permanent disability
Huang et al. [48]	Survey (computer assisted interviews) of workers from a workers’ compensation database Washington State, USA All injury types	*n* = 582 complete interviews Time since claim finalization 11–15 months (mean 12.8 months) Age Mean = 49.13 Sex Female 33%, Male 67%	Safety Climate	At the organizational level, which refers to workers’ perceptions related to the present employer’s top management: Workers returning to work following permanent impairment indicated that perceived safety climate in the workplace was positively related to safety training, autonomy in the workplace, supervisor support and co-worker support Job demands were indicated to have a significant impact on the perceived safety climate of the workplace for workers with permanent impairment At the group level, which refers to workers’ perceptions related to the direct supervisors: For workers returning to work after permanent impairment, safety training, autonomy, supervisor and co-worker support all contributed to perceptions of safety climate Job demands were also negatively associated with safety climate at the group level	1. The shortened version of the safety climate questionnaire is a useful tool for this specific population of workers 2. Further research is recommended to identify ways to improve safety climate for injured workers returning to work following permanent impairment
Larsson and Björnstig [49]	Survey of workers from a workers’ compensation database Umeå, Sweden All reported occupational injuries	*n* = 465 *n* = 58 with permanent medical impairment Time since claim finalization 4.4 years Age < 40 *n* = 302 = > 40 *n* = 163 Sex Female 20%, Male 80%	Symptoms Return to Work/Employment Status	Persistent symptoms were more common in injured workers with a permanent impairment over 5% (pain, reduced strength, limited movement) relative to those with a permanent impairment rating of 5% or less Injured workers with higher permanent impairment rating were more often on long-term sick leave or had retired early	Nil
Martin et al. [50]	Workers’ compensation database cohort with comparisons to the general population West Virginia, USA Low-back injuries	*n* = 14,219 *n* = 4013 with Permanent Disability Time since claim finalization Not defined Age Median 37.7 at time of injury Sex Female 37%, Male 73%	Mortality Return to Work/Employment Status	Overall mortality and mortality from drug overdoses involving opioids within the cohort were higher in injured workers with permanent partial disability, though not permanent total disability When compared to the general population, overall mortality of the cohort was less than that of the general population. This was postulated to represent the health benefits of work. However, for those in the cohort with permanent disability, mortality was the same as the general population, suggesting a low-back injury resulting in permanent disability negates the health benefits of work	1. Work related disability is a significant social burden and should be mitigated through more structured return to work support 2. Research designed to address factors that impact work‐related disability and mortality is recommended to determine associations and modifiable factors
O’Hagan et al. [51]	Survey (via telephone interview) of workers from a workers’ compensation database Ontario, Canada All injury types	*n* = 494 with permanent disabilities Time since claim finalization Not defined Age Mean = 44 Sex Female 61%, Male 39%	HRQoL* Physical Health/Physical Disability Mental Health Personal Relationships Health Care Utilization Wages and Financial Return to Work/Employment Status	Lower education and lower pre-injury personal income were associated with higher levels of psychological distress in the cohort Diagnosis of depression was associated with female sex and younger age Females were more likely to have sleep issues than males Younger age was associated with higher report of concentration problems In a basic side by side comparison of prevalence rates from the general population, injured workers with permanent disabilities following workplace injury are more likely to have diagnosed depression, had higher levels of medication abuse, reported greater inability to concentrate, and had more sleep problems	1. Increased awareness is needed to acknowledge the nature of persistent mental health issues following on from workplace injuries and workers’ compensation claims, as well as the public health impact due to the mental health of injured workers with permanent impairments
Scott-Marshall et al. [53]	Work injury database cohort with comparison to a general population Ontario, Canada All injury types	*n* = 3745 *n* = 537 (14%) with permanent impairment Time since claim finalization Not defined Age Female mean = 36.90 Male mean = 34.41 Sex Female 34%, Male 66%	Personal Relationships	The female injured workers with a permanent impairment from a work injury were 17% less likely to marry relative to individuals without a workers’ compensation related permanent impairment High levels of physical impairment (greater than 10% based on the American Medical Association guidelines) reduced the rate of marriage in female injured workers by 22% compared to control cases Permanent impairment was not shown to impact the probability of marriage in the male injured worker population	1. Further investigation is required to identify system-based strategies to facilitate injured workers’ adjustment across multiple life domains, including social factors like relationship development
Scott-Marshall et al. [52]	Work injury database cohort with comparison to a general population Ontario, Canada All injury types	*n* = 106,208 *n* = 19,322 with permanent impairment Time since claim finalization Not defined Age Female mean = 41.87 Male mean = 43.91 Sex Female 32%, Male 68%	Mortality Wages and Financial Return to Work/Employment Status	The rate of death for male injured workers with permanent impairment from a work injury (14%) was greater than individuals without a work injury related impairment (9%) The rate of death for female injured workers with permanent impairment from a work injury (6%) was greater than individuals without a work injury related impairment (4%) Female injured workers with permanent impairment from a work injury were 30% more likely to die than individuals without a work injury related impairment in the assessed period (up to 19 years after injury). This effect was more pronounced if the permanent impairment occurred at an earlier age or in those with higher levels of work disability (defined by earnings) Similarly, male injured workers with a permanent impairment also had increased risk of death, and this was also more pronounced in those who had a permanent impairment at a younger age, had higher levels of work disability or had higher levels of permanent impairment	1. Further research is required into the long-term effect of work injuries on health that considers physical, psychological and social factors. Important potential psychological factors might include an individual’s sense of self and their ability to cope and adapt 2. Improving rehabilitation services for injured workers to assist injured workers with return to work after injury, is recommended to improve longevity and overall wellbeing
Sears et al. [54]	Survey (computer assisted interviews) of workers from a workers’ compensation database Washington State, USA All injury types	*n* = 598 Time since claim finalization Approximately 1 year Age At time of survey: 19–73 Sex Female 33%, Male 67%	Physical Health/Physical Disability Mental Health Return to Work/Employment Status	Permanent disability after a work injury resulted in significant negative impacts 1 year after claim closure including poorer health and chronic ongoing pain, barriers in continuing employment and negative economic impacts from the injury Specifically: 22% of injured workers with permanent impairment had not returned to any sort of work in the first year after claim closure Injured workers who had returned to work reported that permanent impairment made it difficult to get a job (47%) and to keep their job (58%) A year after claim closure, 66% reported moderate to very severe pain and 40% reported pain interference with work Approximately 13% reported new work injuries; over half thought permanent impairment increased their reinjury risk Injured workers with a higher degree of impairment (≥ 10% whole body impairment) more frequently reported working fewer hours, earning less, and being at higher risk of losing their current job due to their impairment	1. There is need to develop workplace and workers’ compensation system interventions that promote sustained return to work and prevent reinjury for injured workers with permanent impairment 2. This should be considered in the context of an important public health initiative
Sears et al. [55]	Work injury database cohort linked to state wage data Washington State, USA All injury types	*n* = 43,114 No impairment *n* = 74% Impairment < 10% *n* = 19.5% Impairment ≥ 10% *n* = 6.5% Time since claim finalization Not specifically defined 3–11 year range suggested Age 18 and above Sex Female 43%, Male 57%	Physical Health/Physical Disability Reinjury Return to Work/Employment Status	Injured workers with a higher degree of impairment are more likely to be reinjured Based on hours worked, injured workers with ≥ 10% whole body impairment had a 34% higher risk of reinjury relative to injured workers with no permanent partial disability award Injured workers with a permanent impairment who completed an approved vocational rehabilitation program were less likely to be re-injured than those who did not complete the program Similarly, those that opted for self-directed retraining rather than a vocational rehabilitation program were more likely to be re-injured Elevated risk of reinjury is highest in the first 6 months after return to work, but continues up to 4 years where it appears to level off	1. Further efforts are required to understand the mechanisms underlying reduced reinjury rates after undertaking formal vocational rehabilitation programs versus self-directed retraining is needed 2. The first 6 months after return to work may be an important window for managing reinjury
Sears et al. [56]	Work injury database cohort linked to state wage data Washington State, USA All injury types	*n* = 43,968 No impairment *n* = 32,450 Impairment < 10% *n* = 8,604 Impairment ≥ 10% *n* = 2,914 Time since claim finalization Up to 10 years Age At first claim closure 18 and above Sex Female 43%, Male 57%	Physical Health/Physical Disability Wages and Financial Return to Work/Employment Status	Injured workers with higher level of permanent impairment (whole body impairment ≥ 10%) had delayed return to work, shorter average times to first return to work interruption, and higher rates of return to work interruptions and time without wages 27% of injured workers with whole body impairment ≥ 10% WBI did not return to paid work after claim closure The following covariates had strong association with return to work outcomes, though with smaller effect sizes than the level of permanent impairment; Injured workers in the most rural location experience a 34% higher rate of return to work interruption compared to workers in the most urban location Female injured workers were more likely to experience return to work interruptions than male workers Older injured workers were also more likely to experience poorer return to work outcomes than younger workers Higher scores on the Functional Co-morbidity Index was significantly associated with poorer employment outcomes for three of the four modelling approaches. This suggests that, for injured workers with comorbidities, sustained RTW is more challenging than initial RTW Engagement in a structured vocational rehabilitation was associated with better return to work outcomes Self-directed retraining programs were associated with poorer return to work outcomes, compared to conventional retraining programs	1. The workers’ compensation system impacts large numbers of injured workers. Further understanding of the mechanisms which impact return to work outcomes in the workers’ compensation system is needed
Sears et al. [57]	Survey (computer assisted interviews) of workers from a workers’ compensation database Washington State, USA All injury types	*n* = 567 all with permanent impairment Impairment < 10% *n* = 77.6% Impairment ≥ 10% *n* = 22.4% Time since claim finalization 11–15 months (mean 12.8 months) Age 18 and above Sex Female 32%, Male 68%	Return to Work/Employment Status Reinjury	Lower levels of reported support and safety climate were associated with return to work interruption and reinjury 12% of injured workers had been reinjured in their current or most recent job 12% of injured workers were no longer working at the time of interview The most frequently reported reasons for return to work interruption were impairment, disability, and/or pain from the previous work injury Injured workers aged 65 and older had more than 3 times the odds of return to work interruption, compared to workers aged between 18 and 34 Union members had nearly 3 times the odds of reinjury, compared to non-union Workplace factors that were shown to be protective against return to work interruption; Adequate employer/ health care provider communication The injured worker was not stigmatized by their supervisor The injured worker was not stigmatized by their co-workers Co-worker support Workplace factors that were shown to be protective against reinjury: The presence of a health and safety committee Workers are comfortable reporting unsafe situations at work Low job strain Workplace factors that were shown to be protective against both return to work interruption and reinjury: An appropriate organization level safety climate An appropriate group level safety climate Supervisor support Social support An ability to take time off work for personal/family matters	1. Secondary prevention efforts to sustain return to work and prevent reinjury may reduce health, economic, and social burdens of occupational injuries 2. Improving prevention of work place injuries requires consideration of organizational and psychosocial factors which include flexible leave options, safety culture and supervisor support within the work environment
Sears et al. [58]	Qualitative study of workers from a workers’ compensation database (open ended responses to specific questions) Washington State, USA All injury types	*n* = 567 all with permanent impairment Time since claim finalization 11–15 months (mean 12.8 months) Age 19–73 Sex Female 33%, Male 67%	Satisfaction Return to Work/Employment Status Interviews and Open Ended Questioning	All injured workers had returned to work after injury (13% where no longer working at the time of interview) 28% of injured worker study participants reported that no change was needed to the workers’ compensation system, while 58% provided suggestions or critiques The most frequent workers’ compensation system theme, referenced was—reduce delays/simplify process/improve efficiency In regard to potential improvements of the workers’ compensation system (*n* = 335): 60% suggested improvements were needed in efficiencies and access to services, with reducing delays a key theme 35% suggested the need for improved support to navigate the scheme and social support, better communication and increased respect 18% suggested the need for system and legal reforms In regard to potential improvements of the vocational rehabilitation process (*n* = 56): 43% suggested more worker choice input into the vocational training plan would be beneficial as well as providing higher quality and longer durations of services 41% suggested changes to access and efficiency, including more competent rehabilitation counsellors 21% suggested improvements in communication, respect and support navigating through the process	1. There is substantial opportunity for improvement in workers’ experience with the workers’ compensation system 2. Injured workers’ feedback may reflect opportunities to reduce administrative burden and to improve health and RTW outcome
Sears et al. [59]	Work injury database cohort linked to state wage data and Survey (computer assisted interviews) of workers from a workers’ compensation database Washington State, USA All injury types	Cohort *n* = 11,184 Workers ≥ 65 years old accounted for 4.5% (499/11,184) of the cohort sample, and 5.8% (34/582) of the survey sample Time since claim finalization First claim closed between January 1, 2009 through December 31, 2017 Age 18 and above Sex Female 41%, Male 59% Survey *n* = 582 complete interviews Time since claim finalization 11–15 months (mean 12.8 months) Age Mean = 18 and above Sex Female 33%, Male 67%	Reinjury	Using survey data, workers over the age of 65 returning to work after permanent impairment have a twofold risk of reinjury compared to those under the age of 65 Work demands and workplace conditions did not influence the risk of reinjury for workers While there was limited data compared to workers less than 65, workers aged 65 + were: Less often to in full time employment More often in a similar role now to their pre-injury role More often engaged in non-paid work More often not working because of retirement Less certain they would be working in 6 months’ time Less often a union member Less likely to have employer sponsored health insurance Were more likely satisfied with their employment Had a higher prevalence of arthritis and diabetes	1. A call for an increased level of attention to be paid to injury prevention, specifically related to older workers 2. Further research is needed to determine contributing factors to reinjury in older workers with permanent impairment 3. Better understanding is required to determine where injury related medical expenses are applied for older workers above 65 utilizing workers’ compensation and Medicare
Sears et al. [60]	Survey including qualitative study of workers from a workers’ compensation database (open ended responses to specific and open ended questions) Washington State, USA All injury types	*n* = 560 all with permanent impairment Time since claim finalization 11–15 months Age 19–73 (mean = 49.2) Sex Female 32%, Male 68%	Engagement in Workplace Wellness Programs Qualitative Methodology	At the time of interview, 88.2% of workers were in paid employment and 51.4% expressed interest in participating in Workplace Wellness Programs Lower income workers and younger workers were more likely to express interest in participating in Workplace Wellness Programs Workers were more likely to express interest in participating in Workplace Wellness Programs if they experienced: Poorer health status Poorer work functional status More pain More pain interference with work Higher perceived reinjury risk More difficulty getting or keeping a job due to their permanent impairment Experienced reduced income or job security compared to their pre-injury level Did not have health insurance, or who had more chronic health conditions Workers stated reasons for not wanting to engage in Workplace Wellness Programs including; Being too busy Inconvenience Not expecting it to be helpful Workers who were not interested in Workplace Wellness Programs due to being involved in their own fitness and health management, were more likely to have higher income and higher levels of employer-based health insurance	1. Understanding participation barriers to engaging in Workplace Wellness Programs is needed. This may help to determine strategies for facilitating engagement
Sprehe [61]	Clinic cohort survey Florida, USA Injuries that included psychiatric disability	*n* = 108 (*n* = 79 with settled claims) Time since claim finalization Not defined Age Not defined Sex Not defined	Physical Health/Physical Disability Return to Work/Employment Status Symptoms Mortality	Of the 79 settled cases, 62 reported no improvement 46 were not working Of those working, 23 felt their work situation had not improved post-settlement The author rated 76% of the settled cases as not improved overall, commenting that settlement itself does not seem to individual improvement in this cohort	Nil
Tait et al. [62]	Workers’ compensation database cohort who underwent post-settlement interviews Missouri, USA Low-back injuries	*n* = 1472 Time since claim finalization Mean = 21 months (Claims were settled between January 2001 and June 2002) Age 18 and above Sex Female 38%, Male 62%	HRQoL* Symptoms Physical Health/Physical Disability Mental Health Return to Work/Employment Status	A weak, “paradoxical” association was identified whereby people with higher disability ratings reported better outcomes at follow-up With collective analysis of seven post-settlement outcomes (pain intensity, general physical health, general mental health, pain catastrophising, pain-related disability, full-time employment, part-time employment), disability rating only shared 3% of its variance with these outcomes	1. The disparities demonstrated in post-settlement outcomes compared with levels of disability ratings, may indicate that disability rating methodology should be re-evaluated in the context of workers’ compensation claims
Tait and Chibnall [63]	Workers’ Compensation database cohort who underwent post-settlement interviews coupled with state judicial records Missouri, USA Low-back injury	*n* = 1345 Time since claim finalization 5 years Mean = 20.8 months Age Mean = 43.2 Sex Female 38%, Male 62%	Legal Proceedings	There was increased involvement in legal cases in the immediate 5 years after people had finalized a claim, compared to the preceding 5 years Legal proceedings related to financial cases increased over the 5 years post-settlement, in a linear pattern. This may suggest an increase in financial burden over this period General and domestic financial cases were more likely to involve African-American, compared to non-Hispanic white injured workers Injured workers with low-back injury who are 35 or younger at the time of settlement are 14% more likely to be involved in legal proceeding of a financial nature after settlement, compared to older injured workers Injured workers with lower settlement awards are involved in more legal proceedings, of any type, in the 5 years after settlement than those with larger settlement awards	1. Workers’ compensation administrative systems require review to facilitate better adjustment to life for injured workers following settlement of their workers’ compensation claim
Tait and Chibnall [64]	Workers’ Compensation database cohort who underwent post-settlement interviews Missouri, USA Low-back injury	*n* = 374 (*n* = 358 with complete data at both follow-ups) Time since claim finalization 21 months at first follow-up and 72 months at second follow-up (Claims were settled between January 2001 and June 2002) Age No Disability Group Mean = 42.3 Early Disability Group Mean = 47.7 Late Disability Group Mean = 46.3 Sex Female 39%, Male 61%	Satisfaction Symptoms Physical Health/Physical Disability Return to work/Employment status	64% of injured workers had continued to work throughout the follow-up period 21% were considered to have early disability and 15% late disability with regard to work status post-permanent impairment The following factors were associated with early disability after permanent impairment provision: Receipt of temporary total disability benefits African-American race Dissatisfaction with medical care Dissatisfaction with employer Larger monetary settlement at claim closure Lower education levels Lower compensation rate Older age Late disability was associated with: Receipt of temporary total disability benefits African-American race Older age	1. Perception of injustice should be a measure utilized in further research in workers’ compensation 2. Better understanding of how perceived injustice influences long-term outcomes for injured workers following settlement is needed to reduce societal costs
Yassi [65]	Workers’ compensation database cohort with file reviews and surveys Ontario, Canada Occupational allergic respiratory disease	Baseline *n* = 154 (*n* = 43 with permanent disability) Follow-Up *n* = 40 (*n* = 19 with permanent disability) Time since claim finalization Not defined Age Mean = 39.55 Sex Female 28%, Male 72%	Physical Health/Physical Disability Return to Work/Employment Status Symptoms	100% of injured workers with permanent disability at follow-up (*n* = 19) had ongoing symptoms, compared to 70% of those without permanent disability While still symptomatic 58% of the permanent disability at follow-up reported ongoing improvement, compared to 95% of those without permanent disability Half of injured workers who had received permanent disability awards, were unemployed at the time of follow-up	1. Review of medical records and/or low-up patient survey might be useful for further research in this field 2. Promotion of cleanliness in the workplace in relation to hazard chemicals should be a priority

*HRQol health-related quality of life

### Study Characteristics

Of the included articles, 23/32 articles were cohort studies, 4/32 were case series, 3/32 were cross-sectional studies, and 2/32 were qualitative (via open ended questions on a survey) studies. Use of population databases for comparators was common. Twenty-one articles were from the United States of America, six from Canada, three from Australia, one from Sweden, and one from Taiwan. In respect to types of injuries evaluated in the studies, 16/32 included all injury types, 11/32 were low-back injuries, 1/32 included back or neck injuries, 1/32 included carpal tunnel syndrome, dermatitis and upper extremity fractures, 1/32 included major physical trauma, 1/32 evaluated injuries that included psychiatric disability and 1/32 included occupational allergic respiratory disease.

### Participant Characteristics

Twenty-five articles defined the time since claim finalization, ranging from 1 month [41] to 15 years [51] post-claim. Most articles reported a large age range that covered the entirety of the expected working ages. Of those reporting the mean age, the mean was between 34 and 49 years. Of the 30 articles that defined the sex of participants, female participants comprised on average 39% and male participants 61% of the sample.

### Measures of Wellbeing

No studies were identified that included a specific wellbeing measure. The included articles refer to specific post-claim finalization measures that are contributory constructs of wellbeing [70, 71]. The most common constructs, include return to work and employment status (19/32 articles), physical health/disability (15/32 articles), ongoing symptoms (11/32 articles), and mental health (11/32 articles) and are presented in Appendix 2 and summarized in Appendix 3.

### Post-Claim Finalization Outcomes

In general, people who were injured at work had poorer long-term outcomes when compared to those who were not injured at work [43, 51, 54–57, 69]. Injured workers with permanent impairments following workplace injury were more likely to have an unstable work status, higher risk of reinjury, ongoing symptoms, and an increased risk of mortality [47, 51, 57–61, 65]. Injured workers were more likely to experience ongoing chronic health conditions than the general population [43, 58, 64]. Injured workers with permanent disability were more likely to be diagnosed with depression [43, 55]; however, one study [50] indicated that settling a claim had a positive effect on the SF-36 Mental Component Summary score compared to having an unsettled claim.

### Recommendations from the Included Articles

**Fig. 3:**
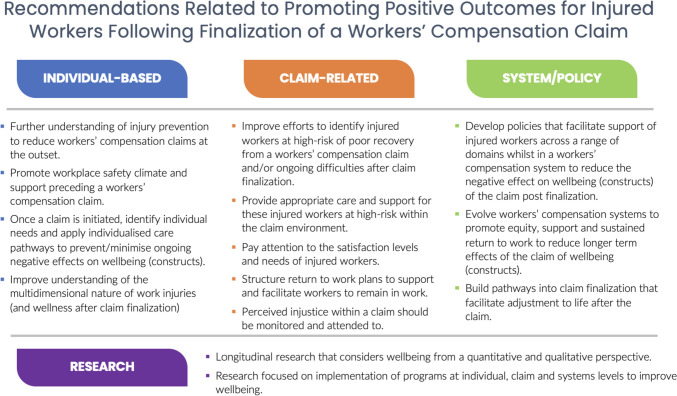
Recommendations from the literature review related to post-claim finalization outcomes

Recommendations from the included articles are outlined in Table 1, with a synthesis presented in Fig. 3. Broadly these recommendations fall into three categories: Individual-related, for example further understanding of injury prevention to reduce workers’ compensation claims at the outset and promotion of workplace safety climate, claim-related, for example improving efforts to identify people at high-risk of poor recovery from a workers’ compensation claim, and system/policy related recommendations, for example developing policies to support injured workers across a range of domains while in a workers’ compensation system to reduce the negative effect on wellbeing. Research recommendations included more longitudinal research that considers wellbeing from a quantitative and qualitative perspective, as well as research focused on implementation of programs at individual, claim, and systems’ levels to improve wellbeing.

### Critical Appraisal

Results of the critical appraisal are presented in Appendices 5–8.

## Discussion

### Main Findings

This scoping review identified 32 articles that assessed aspects of wellbeing in injured workers who had finalized a workers’ compensation claim and synthesized knowledge on this topic. No study specifically measured overall wellbeing as an independent construct through the use of specific measures [72–74]. One article did refer to a concept of ‘long term adjustment’ as an umbrella term encompassing individual measures of pain, disability, catastrophising, long-term unemployment, and reliance on social security [31]. This concept of long-term adjustment might be akin to the concept of wellbeing. More generally, injured workers who had finalized a workers’ compensation claim had ongoing negative consequences from the claim at an individual level [43, 51, 54–57, 69]. When comparisons were made to the general population, injured workers post-claim appeared disadvantaged in multiple ways which arguably reflect reduced wellbeing and greater cost burden to their communities [43, 47, 51, 57–61, 64, 65]. It is important to note though that most of the cases in this review had finalization of their claim defined by permanent impairment/disability (for a musculoskeletal complaint), so by definition of permanent impairment/disability, injured workers in these cases are likely to experience ongoing issues related to physical health.

### Strengths and Limitations

This study utilized a robust design and procedure informed by established frameworks and reporting standards for scoping reviews [35–37]. This included using the services of a librarian, searching multiple database repositories from broad subject areas and using multiple investigators in the literature review and data extraction processes. The research team included academics and practitioners with broad research and clinical experience in workers’ compensation, occupational medicine, physical rehabilitation, law, and wellbeing. This mix of contributors provided a breadth of knowledge and interpretation befitting the nature of the review.

A limitation may have been restricting the scope of the hand searching and online literature search to local regulatory websites and handsearching of reference lists of the included articles. The majority of articles were from North American workers’ compensation schemes and may not be representative of other parts of the world. The wide date range of the final included papers is representative of the current literature base. However, potential changes in workers’ compensation system over this time period were not considered in the review. Further research might investigate the effect of legislative change on worker outcomes. There is no universal language for finalization of a claim, and our definition of this concept might have potentially limited the search. Further, with most of the included studies being defined by permanent impairment/disability, the findings might not reflect what occurs after claim finalization where most claims are of short duration with minimal disruption to work [75]. Nevertheless, future burden to society is more likely to result from finalized claims with a permanent impairment awarded and/or when there has been significant time off work.

### What Does the Current Literature Base Indicate?

It is well documented that a workers’ compensation claim can have significant negative effects on some injured workers during a claim [14, 15, 23, 76]. The findings of this scoping review support the proposition that this burden on injured workers can continue after claim finalization. These findings are contrary to earlier medico-legal assertions, often frequently cited by insurers that the finalization of a workers’ compensation claim will result in positive outcomes for all injured workers [77–81]. While this review did not identify any studies directly assessing wellbeing, it did identify a range of impacts on physical and psychosocial or behavioural health post-claim. Contemporary practice for the management of injured workers during a compensation claim demands a biopsychosocial approach [25, 82], to maximize integration back into work. The progression over recent decades towards workers’ compensation systems that promote workplace rehabilitation and wider ranging health supports (including support for mental health) beyond financial compensation is in keeping with the biopsychosocial approach [83]. Injured workers who were engaged in structured vocational rehabilitation and had supportive workplace environments during their claim were more likely to experience less interruptions to work post-claim [59, 61]. This supports the importance of a biopsychosocial approach to claim management and the transition back to life after claim finalization. Similarly, the ongoing management of people following a claim is likely to need broad biopsychosocial approaches.

Wellbeing has increasingly become a favoured term and metric associated with government policy making and planning [38, 84, 85] (Fig. 1), and this may help quantify the nature of the economic burden that results from workers’ compensation claims. Disruption of individual wellbeing during a claim that endures beyond claim finalization may have societal implications. Cost shifting (from workers’ compensation schemes to other public services) may be one issue. For example, when financial support provided under a compensation claim is withdrawn (though the claim is not closed), people may go on to receive social security payments [26, 28] and publicly provided health care [27]. Further issues may include ongoing deterioration of personal relationships and social networks. The findings of this scoping review indicate that this broader societal impact conflicts with the concepts of a wellbeing economy that promotes “dignity, connection, fairness, and participation” [11] (p. 5). However, the ongoing burden and potential cost-shifting following the end of a claim may be difficult to quantify as people transition from well-monitored compensation schemes [3, 4] to environments where the lasting impact on wellbeing due to the claim is not monitored. Consequently, wellbeing may be a valuable collective measure of these impacts for individuals and the broader community.

### Future Recommendations

**Fig. 4:**
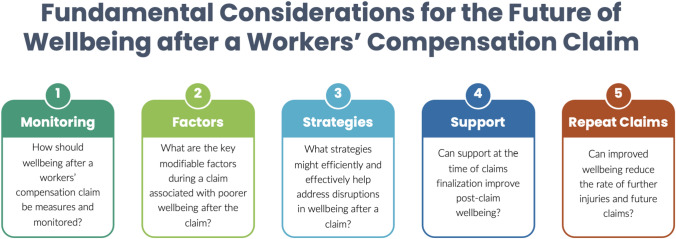
Fundamental considerations for the future of wellbeing following claim finalization

Addressing wellbeing directly could have positive effects for the individual and society as a whole. This should be a consideration during a claim, at the time of finalization and beyond finalization. A broad array of recommendations at individual, claim, and system/policy levels, have been identified in this scoping review (Fig. 3). It may be pertinent for researchers, schemes, and service providers to consider global initiatives to which these recommendations align (Fig. 4). Increased understanding of individual wellbeing following the end of a workers’ compensation claim is needed. Ultimately this might guide scheme reforms and assist with the development of programs and resources for enhancing wellbeing following finalization of a workers’ compensation claim.

The workers’ compensation environment has been demonstrated to be an important environment for implementation of interventions and strategies to facilitate outcomes within the life time of the claim [48, 86–90]. Further understanding and measurement of these outcomes beyond the end-point of the claim may help determine if these outcomes continue, and whether these outcomes influence individual wellbeing and broader society following claim finalization. In addition, there was a notable absence in consideration of the lived experience, beliefs, and attitudes of injured workers around this critical transition period. Individuals’ beliefs and attitudes are considered to be an integral part of broader behavioural health [91]. A better understanding of the beliefs and attitudes of injured workers may facilitate a better understanding of the needs of these individuals and may provide a compass for developing and implementing person-centred support. It is not clear with whom the responsibility lies for collecting and evaluating this data and clarifying this duty is considered an important step in further research.

Transitional support for injured workers following claim finalization is an important consideration that has been implemented and reviewed in this population but has not been evaluated in depth in the existing literature base [26–28]. Understanding whether or not transitional support influences the wellbeing of injured workers following the finalization of a claim may facilitate further decision making regarding the most appropriate pathways to support injured workers through this process. Furthermore, there is not a clear understanding of the influence of multiple or recurrent claims on the wellbeing of injured workers following claim finalization. This was previously identified as an understudied phenomenon in the occupational literature [48]. Knowledge in this area may also guide designation of appropriate support measures that are specifically tailored to the needs of injured workers.

## Conclusion

This scoping review has synthesized a body of literature that evaluates outcomes of injured workers following the end of a workers’ compensation claim. Disruption to wellbeing as a result of engaging in a workers’ compensation claim appears to have implications that last well beyond claim finalization. There appears to be potential for ongoing burden for individuals, employers, and society after finalization of a workers’ compensation claim. Further research that examines the specific nature of wellbeing after finalization of a workers’ compensation claim is warranted.

## Supplementary Information

Below is the link to the electronic supplementary material.

Supplementary file1 (DOCX 83 KB)
