# Supplementary material for: Wellbeing After Finalization of a Workers’ Compensation Claim: A Systematic Scoping Review
Source: J Occup Rehabil. 2024 Jan 30;34(4):717–39. doi: 10.1007/s10926-023-10168-6 (PMC11550234; doi:10.1007/s10926-023-10168-6)
Supplement: Supplementary file 1 — Supplementary file1 (DOCX 83 KB) [file 10926_2023_10168_MOESM1_ESM.docx]

**Appendix 1: Search Strategy for Scoping Review**

| "Work* Comp*" OR "Injury Claim" OR "Compensation claim*" OR "Work* Injur*" OR "Work related injur*" OR "Compensation scheme" OR "Injur* Compensation" OR "Employer's indemnity" OR "Work* Indemnity" OR "Occupational accident insurance" OR "Occupational accident claim*" OR "Occupational injury insurance" OR "Occupational injury claim*" OR "Occupational indemnity" OR “Occupational Injury” OR "Industrial injury" OR "Work* Liability Insurance" OR “Injured Worker” OR “employ* indemnity” |
| --- |
| AND |
| Finalisation OR Finalization OR Settlement OR "post settlement" OR post-settlement OR (Claim AND (end OR settl* OR termination OR closure)) OR "End of claim*" OR "Inactive claim" OR "Lump sum" OR “commutation” OR “Redemption” OR “Permanent Impairment” OR “Permanent Disability” OR “Permanent Partial Disability” OR “Closure” |
| AND |
| Wellbeing OR Wellness OR Well-being OR Recovery OR "Quality of Life" OR "Return to work" OR "Psychological distress" OR "psychosocial factor*" OR satisfaction OR health |

**Appendix 2: Frequency of wellbeing constructs evaluated following claim finalisation (of 32 included articles)**

**Appendix 3: Constructs evaluated in the literature relating to wellbeing**

| **Construct** | **Studies** | **Specific Tool** | **Number of Studies** |
| --- | --- | --- | --- |
| **HRQoL** | Chibnall 2010  Harris 2008  O’Hagan 2012  Tait 2006 | SF-12  SF-36 | 2  1 |
| **Symptoms** | Balla 1970  Casey 2017  Chibnall 2005a (race)  Chibnall 2009  Chibnall 2010  Greenough 1989  Larsson 1995  Sprehe 1984  Tait 2006  Tait 2016  Yassi 1988 | Pain Disability Index  Modified Somatic Perception Questionnaire  Pain Intensity | 3  1  4 |
| **Physical Health/Physical Disability** | Casey 2017  Chibnall 2005a  Chibnall 2005b  Edmonds 2021  Foley 2015  Greenough 1989  Harris 2008  O’Hagan 2012  Sears 2021a  Sears 2021b  Sears 2021c  Sprehe 1984  Tait 2006  Tait 2016  Yassi 1988 | Functional Comorbidity Index  Pain Disability Index  Quick DASH  SF-36  SF-12 Physical  Oswestry Disability Index  Degree of Impairment | 1  2  1  1  2  1  3 |
| **Mental Health** | Casey 2017  Chibnall 2005a  Chibnall 2009  Chibnall 2010  Evans 2001  Foley 2015  Greenough 1989  Harris 2008  O’Hagan 2012  Sears 2021a (Workforce)  Tait 2006 | CES-D  Pain Catastrophising Scale  Hospital Anxiety Scale  Hospital Depression Scale  SF-12 Mental  Zung Depression Scale  Axis I Diagnosis  Axis II Diagnosis  SF-36  Functional Comorbidity Index | 1  4  1  1  3  1  1  1  1  1 |
| **Mortality** | Ho 2006  Martin 2020  Scott-Marshall 2014  Sprehe 1984 | EYLL (Estimated Years of Life Lost)  YPLL (Years of Potential Working Life Lost)  NIOSH Life Table Analysis System | 1  1  1 |
| **Return to Work / Employment Status** | Balla 1970  Chibnall 2006  Edmonds 2021  Evans 2001  Foley 2015  Greenough 1989  Larsson 1995  Martin 2020  O’Hagan 2012  Scott-Marshall 2014  Sears 2021a (Workforce)  Sears 2021b (Estimating)  Sears 2021c (Initial return)  Sears 2021d (Workplace)  Sears 2021e (Appraisal)  Sprehe 1984  Tait 2006  Tait 2016  Yassi 1988 | None identified |  |
| **Re-injury** | Edmonds 2021  Sears 2021b (Estimating)  Sears 2021d (Workplace)  Sears 2022a | None identified |  |
| **Personal Relationships** | Foley 2015  O’Hagan 2012  Scott-Marshall 2013 | None identified |  |
| **Health Care Utilisation** | Evans 2001  O’Hagan 2012 | None identified |  |
| **Legal Proceedings** | Chibnall 2010  Tait 2011 | None identified |  |
| **Wages and Financial** | Ballantyne 2016  Chibnall 2005a  Chibnall 2005b (disparities)  Edmonds 2021  Foley 2015  O’Hagan 2012  Scott-Marshall 2014  Sears 2021c (Initial) | LICO (Low Income Cut-Off)  LIM (Low Income Measure) | 1  1 |
| **Satisfaction** | Balla 1970  Chibnall 2005b (disparities)  Chibnall 2010  Sears 2021e (Appraisal)  Tait 2016 | None identified |  |
| **Interviews and Open Ended Questioning** | Sears 2021e  Sears 2022b | None identified |  |
| **Safety Climate** | Huang 2022 | Zohar and Luria’s 32 Item Safety Climate scale (shortened version) | 1 |
| **Workplace Wellness Programs** | Sears 2022b | None identified |  |

**Appendix 4: Common themes presented in the literature related to workers’ compensation outcomes for individuals**

| **Theme** | **Studies that reflect this theme** |
| --- | --- |
| **Return to work and sustained return to work is important for individuals and society** | Edmonds 2021  Martin 2020  Sears 2021a  Sears 2021b  Sears 2021c |
| **Return to work support is important** | Ballantyne 2016  Edmonds 2021  Foley 2015  Huang 2022  Johnson 1998  Martin 2020  Sears 2021b |
| **Workers’ satisfaction in the workers’ compensation claim process is important** | Chibnall 2005  Sears 2021e  Tait 2016 |
| **Workplace injuries and outcomes can be multi-factorial** | Balla 1970  Casey 2017  Chibnall 2006  Chibnall 2010  Evans 2001  Foley 2015  Harris 2008  O’Hagan 2012  Scott-Marshall 2014  Tait 2006  Tait 2016 |
| **Workplace injuries and outcomes can have a significant societal impact** | Ballantyne 2016  Edmonds 2021  O’Hagan 2012  Martin 2020  Scott-Marshall 2014  Sears 2021  Sears 2021d  Tait 2011  Sears 2022b |

**Appendix 5: Joanna Briggs Institute critical appraisal checklist for cohort studies**

Y=Yes

N=No

U=Unclear

NA=Not Applicable

| **Article** | **Were the two groups similar and recruited from the same population?** | **Were the exposures measured similarly to assign people to both exposed and unexposed groups?** | **Was the exposure measured in a valid and reliable way?** | **Were confounding factors identified?** | **Were strategies to deal with confounding factors stated?** | **Were the groups/participants free of the outcome at the start of the study (or at the moment of exposure)?** | **Were the outcomes measured in a valid and reliable way?** | **Was the follow up time reported and sufficient to be long enough for outcomes to occur?** | **Was follow up complete, and if not, were the reasons to loss to follow up described and explored?** | **Were strategies to address incomplete follow up utilised?** | **Was appropriate statistical analysis used?** |
| --- | --- | --- | --- | --- | --- | --- | --- | --- | --- | --- | --- |
| **(Author and Year)** |  |  |  |  |  |  |  |  |  |  |  |
| **Ballantyne et al [2]**  2016 | Y | Y | Y | Y | Y | Y | Y | Y | Y | NA | Y |
| **Casey and Ballantyne [3]** | Y | Y | Y | Y | Y | Y | Y | Y | Y | NA | Y |
| 2017 |  |  |  |  |  |  |  |  |  |  |  |
|  |  |  |  |  |  |  |  |  |  |  |  |
|  |  |  |  |  |  |  |  |  |  |  |  |
| **Chibnall et al [4]**  2005a | Y | Y | Y | N | NA | Y | Y | Y | Y | NA | Y |
| **Chibnall and Tait [5]**  2005b  Disparities | Y | Y | Y | N | NA | Y | Y | Y | Y | NA | Y |
| **Chibnall et al [6]**  2006 | Y | Y | Y | N | NA | Y | Y | Y | Y | NA | Y |
| **Chibnall and Tait [7]**  2009 | Y | Y | Y | N | NA | Y | Y | Y | Y | NA | Y |
| **Chibnall and Tait [8]**  2010 | Y | Y | Y | N | NA | Y | Y | Y | Y | NA | Y |
| **Edmonds et al [9]**  2021 | N | Y | Y | N | NA | Y | Y | Y | Y | NA | Y |
| **Evans et al [10]**  2001 | Y | Y | Y | N | NA | Y | Y | Y | Y | NA | Y |
| **Ho et al [14]**  2006 | Y | Y | Y | N | NA | Y | Y | Y | Y | NA | Y |
| **Huang [15]**  2022 | NA | NA | Y | N | NA | Y | Y | Y | Y | Y | Y |
| **Larsson and Björnstig [16]**  1995 | Y | Y | Y | N | NA | Y | Y | Y | Y | NA | Y |
| **Martin et al [17]**  2020 | Y | Y | Y | N | NA | Y | Y | Y | Y | NA | Y |
| **Scott-Marshall et al [20]**  2013 | Y | Y | Y | Y | Y | Y | Y | Y | Y | NA | Y |
|  |  |  |  |  |  |  |  |  |  |  |  |
| **Scott-Marshall et al [19]**  2014 | Y | Y | Y | Y | Y | Y | Y | Y | Y | NA | Y |
|  |  |  |  |  |  |  |  |  |  |  |  |
| **Sears et al [22]**  2021b | NA | NA | Y | Y | Y | N | Y | Y | Y | NA | Y |
| Estimating time to reinjury… |  |  |  |  |  |  |  |  |  |  |  |
| **Sears et al [23]**  2021c | Y | Y | Y | Y | Y | Y | Y | Y | Y | NA | Y |
|  |  |  |  |  |  |  |  |  |  |  |  |
|  |  |  |  |  |  |  |  |  |  |  |  |
| **Sears et al**  **[24]**  2021d | NA | NA | Y | Y | Y | N | Y | Y | Y | NA | Y |
| **Sears et al [26]**  2022a | Y | Y | Y | Y | Y | Y | Y | Y | Y | Y | Y |
| **Tait et al [29]**  2006 | Y | Y | Y | N | NA | Y | Y | Y | Y | NA | Y |
| **Tait and Chibnall [30]**  2011 | Y | Y | Y | N | NA | Y | Y | Y | Y | NA | Y |
| **Tait and Chibnall [31]**  2016 | Y | Y | Y | N | NA | Y | Y | Y | Y | NA | Y |
| **Yassi [32]**  1988 | NA | NA | N | N | NA | Y | N | Y | Y | NA | Y |

**Appendix 6: Joanna Briggs Institute critical appraisal checklist for analytical cross sectional studies**

Y=Yes

N=No

U=Unclear

NA=Not Applicable

| **Article** | **Were the criteria for inclusion in the sample clearly defined?** | **Were the study subjects and the setting described in detail?** | **Was the exposure measured in a valid and reliable way?** | **Were objective, standard criteria used for measurement of the condition?** | **Were confounding factors identified?** | **Were strategies to deal with confounding factors stated?** | **Were the outcomes measured in a valid and reliable way?** | **Was appropriate statistical analysis used?** |
| --- | --- | --- | --- | --- | --- | --- | --- | --- |
| **(Author and Year)** |  |  |  |  |  |  |  |  |
| **Foley and Silverstein [11]**  2015 | Y | Y | Y | Y | Y | Y | Y | Y |
| **O’Hagan et al [18]** | Y | Y | Y | Y | Y | Y | Y | Y |
| 2012 |  |  |  |  |  |  |  |  |
| **Sears et al [21]** |  |  |  |  |  |  |  |  |
| 2021a | Y | Y | Y | Y | Y | Y | Y | Y |
|  |  |  |  |  |  |  |  |  |
| Workforce reintegration… |  |  |  |  |  |  |  |  |

**Appendix 7: Joanna Briggs Institute critical appraisal checklist for qualitative studies**

Y=Yes

N=No

U=Unclear

NA=Not Applicable

| **Article** | **Is there congruity between the stated philosophical perspective and the research methodology?** | **Is there congruity between the research methodology and the research question or objectives?** | **Is there congruity between the research methodology and the methods used to collect data?** | **Is there congruity between the research methodology and the representation and analysis of data?** | **Is there congruity between the research methodology and the interpretation of results?** | **Is there a statement locating the researcher culturally or theoretically?** | **Is the influence of the researcher on the research, and vice- versa, addressed?** | **Are participants, and their voices, adequately represented?** | **Is the research ethical according to current criteria or, for recent studies, and is there evidence of ethical approval by an appropriate body?** | **Do the conclusions drawn in the research report flow from the analysis, or interpretation of the data?** |
| --- | --- | --- | --- | --- | --- | --- | --- | --- | --- | --- |
| **(Author and Year)** |  |  |  |  |  |  |  |  |  |  |
| **Sears et al [25]** | Y | Y | Y | Y | Y | Y | Y | Y | Y | Y |
| 2021e |  |  |  |  |  |  |  |  |  |  |
| Appraisal… |  |  |  |  |  |  |  |  |  |  |
| **Sears et al [27]**  2022b | Y | Y | Y | Y | Y | Y | Y | Y | Y | Y |

**Appendix 8: Joanna Briggs Institute critical appraisal checklist for case series studies**

Y=Yes

N=No

U=Unclear

NA=Not Applicable

| **Article**  **(Author and Year)** | **Were there clear criteria for inclusion in the case series?** | **Was the condition measured in a standard, reliable way for all participants included in the case series?** | **Were valid methods used for identification of the condition for all participants included in the case series?** | **Did the case series have consecutive inclusion of participants?** | **Did the case series have complete inclusion of participants?** | **Was there clear reporting of the demographics of the participants in the study?** | **Was there clear reporting of clinical information of the participants?** | **Were the outcomes or follow up results of cases clearly reported?** | **Was there clear reporting of the presenting site(s)/clinic(s) demographic information?** | **Was statistical analysis appropriate?** |
| --- | --- | --- | --- | --- | --- | --- | --- | --- | --- | --- |
| **Balla and Moraitis [1]**  1970 | Y | Y | N | N | N | Y | Y | N | N | N |
| **Greenough and Fraser [12]**  1989 | Y | Y | Y | Y | Y | N | N | Y | N | Y |
| **Harris et al [13]**  2008 | Y | Y | Y | Y | Y | Y | Y | Y | Y | Y |
| **Sprehe [28]**  1984 | Y | Y | Y | Y | Y | Y | N | Y | N | N |

**Appendix 9: Preferred Reporting Items for Systematic reviews and Meta-Analyses extension for Scoping Reviews (PRISMA-ScR) Checklist**

| **Section** | **Item** | **PRISMA-ScR Checklist Item** |
| --- | --- | --- |
| TITLE |  |  |
| Title | 1 | Identify the report as a scoping review. |
| ABSTRACT |  |  |
| Structured Summary | 2 | Provide a structured summary that includes (as applicable): background, objectives, eligibility criteria, sources of evidence, charting methods, results, and conclusions that relate to the review questions and objectives. |
| INTRODUCTION |  |  |
| Rationale | 3 | Describe the rationale for the review in the context of what is already known. Explain why the review questions/objectives lend themselves to a scoping review approach. |
| Objectives | 4 | Provide an explicit statement of the questions and objectives being addressed with reference to their key elements (e.g., population or participants, concepts, and context) or other relevant key elements used to conceptualize the review questions and/or objectives. |
| METHODS |  |  |
| Protocol and registration | 5 | Indicate whether a review protocol exists; state if and where it can be accessed (e.g., a Web address); and if available, provide registration information, including the registration number. |
| Eligibility criteria | 6 | Specify characteristics of the sources of evidence used as eligibility criteria (e.g., years considered, language, and publication status), and provide a rationale. |
| Information sources | 7 | Describe all information sources in the search (e.g., databases with dates of coverage and contact with authors to identify additional sources), as well as the date the most recent search was executed. |
| Search | 8 | Present the full electronic search strategy for at least 1 database, including any limits used, such that it could be repeated. |
| Selection sources of evidence | 9 | State the process for selecting sources of evidence (i.e., screening and eligibility) included in the scoping review |
| Data charting process | 10 | Describe the methods of charting data from the included sources of evidence (e.g., calibrated forms or forms that have been tested by the team before their use, and whether data charting was done independently or in duplicate) and any processes for obtaining and confirming data from investigators. |
| Data items | 11 | List and define all variables for which data were sought and any assumptions and simplifications made. |
| Critical appraisal of individual sources of evidence | 12 | If done, provide a rationale for conducting a critical appraisal of included sources of evidence; describe the methods used and how this information was used in any data synthesis (if appropriate). |
| Synthesis of results | 13 | Describe the methods of handling and summarizing the data that were charted. |
| RESULTS |  |  |
| Selection of sources of evidence | 14 | Give numbers of sources of evidence screened, assessed for eligibility, and included in the review, with reasons for exclusions at each stage, ideally using a flow diagram. |
| Characteristics of sources of evidence | 15 | For each source of evidence, present characteristics for which data were charted and provide the citations. |
| Critical appraisal within sources of evidence | 16 | If done, present data on critical appraisal of included sources of evidence (see item 12). |
| Results of individual sources of evidence | 17 | For each included source of evidence, present the relevant data that were charted that relate to the review questions and objectives. |
| Synthesis of results | 18 | Summarize and/or present the charting results as they relate to the review questions and objectives. |
| DISCUSSION |  |  |
| Summary of evidence | 19 | Summarize the main results (including an overview of concepts, themes, and types of evidence available), link to the review questions and objectives, and consider the relevance to key groups. |
| Limitations | 20 | Discuss the limitations of the scoping review process. |
| Conclusions | 21 | Provide a general interpretation of the results with respect to the review questions and objectives, as well as potential implications and/or next steps. |
| FUNDING |  |  |
| Funding | 22 | Describe sources of funding for the included sources of evidence, as well as sources of funding for the scoping review. Describe the role of the funders of the scoping review. |
